# Reductions in Complex Mismatch Negativity to Extra Tone Gestalt Pattern Deviance in First-Episode Schizophrenia

**DOI:** 10.3389/fpsyt.2020.00505

**Published:** 2020-06-08

**Authors:** Dean F. Salisbury, Brian A. Coffman, Sarah M. Haigh

**Affiliations:** Clinical Neurophysiology Research Laboratory, Department of Psychiatry, Western Psychiatric Hospital, University of Pittsburgh School of Medicine, Pittsburgh, PA, United States

**Keywords:** mismatch negativity, schizophrenia, first episode psychosis, social functioning, biomarker

## Abstract

Although “simple” mismatch negativity (sMMN) to stimulus parameter changes is robustly reduced in long-term schizophrenia (Sz), it is much less reduced in individuals at their first psychotic episode in the schizophrenia-spectrum (FESz). “Complex” MMN (cMMN) reflecting pre-attentive acoustic pattern analysis is also markedly reduced in Sz, but is little studied in FESz. The computational complexity of pattern analysis reflected in cMMN may more greatly stress auditory processing, providing a more sensitive measure of auditory processing deficits at first break. If so, cMMN would provide information about the underlying pathophysiology early in disease course, and may serve as a biomarker for pathology in the Sz prodrome. Twenty-two FESz individuals were compared to 22 volunteer healthy controls (HC) on sMMN and cMMN tasks. For sMMN, pitch- and duration-deviants were presented among standard repetitive tones. For cMMN, repeated groups of 3 identical tones were presented with occasional (14%) groups including an extra identical 4th tone deviant. FESz did not show reductions of pitch-deviant (Cohen's d = 0.08) or duration-deviant MMNs (d =-0.02), but showed large reduction in extra-tone cMMN (d = 0.83). Reduced cMMN was associated with poor social functioning. Reduction in cMMN but not in sMMNs in FESz suggests impairments in late perceptual pattern processing. cMMN is sensitive to subtle pathology and functioning early in disease course which may, in turn, impact social functioning. Future studies in clinical high risk individuals are needed to determine whether this putative biomarker of disease presence is sensitive to the prodromal stage of schizophrenia.

## Introduction

The auditory mismatch negativity (MMN), a neurophysiological measure related to automatic deviance detection, has engendered much research in clinical neuroscience to understand the basic pathophysiology of schizophrenia due to its severe impairment in long-term schizophrenia (Sz). The MMN elicited by infrequent changes in acoustic parameters, such as pitch-deviants (pMMN) and duration-deviants (dMMN), show effect sizes (Cohen's d) in Sz between 0.8 and 1.2 (1, 2). However, this simple parameter MMN (sMMN) is not as reduced in individuals at their first episode of schizophrenia (FESz) (3–6). Recent meta-analyses suggest a dMMN effect size in FESz of 0.4 to 0.5 (2, 7) (which dropped to 0.36 when only studies that matched premorbid IQ or education were included) and a negligible effect size for pMMN of <0.04. Thus, sMMN does not appear to be as sensitive to the pathophysiology early in disease course as it is in later stages.

Like in FESz, sMMN reductions have been, at best, equivocal in clinical high risk for psychosis (CHR) individuals and in truly prodromal individuals within the CHR state. Despite some promising reports of greater dMMN reductions in CHR individuals that converted to psychosis (8, 9), replication has been difficult. For example, Patricia Michie's group originally reported smaller dMMN in a group of 6 CHR individuals that later transitioned to psychosis (10), but recently reported no dMMN reduction in 7 CHR individuals who transitioned to psychosis; in fact those who transitioned to psychosis evinced larger dMMN than those that did not (11). There have also been reports of dMMN being larger in CHR individuals, e.g., (12), although these were very young individuals. In Erickson et al's meta-analysis (2), CHR studies showed an effect size of d = 0.4, again a relatively small effect, with close to chance probability that a CHR individual selected at random would show a MMN worse than the healthy mean (61%).

Thus, sMMN reductions appear to be smaller in early psychosis. Given that sMMN is quite reduced in long-term illness, is related to the underlying circuit pathophysiology (13), and shows progressive reductions after psychosis onset that correlate with longitudinal gray matter loss (5), it is presumed that such auditory pathophysiology is present to a lesser degree early in the psychosis course and prior to the emergence of psychosis. If so, a more computationally-demanding MMN measure may be sensitive to this subtle early disease course pathophysiology. While different etiologies with different pathophysiology may be related to the symptom heterogeneity in schizophrenia at first episode, nearly all patients will show MMN reduction after a few years of psychosis (5). In that most FESz individuals have little sMMN reduction at first episode but develop deficits with disease course, there appears to be a common auditory pathology associated with sMMN across possible differential etiologies. If so, it is possible that a variation of the standard mismatch task may reveal subtle pathology to which sMMN is insensitive.

Healthy individuals produce MMN to a variety of deviants in complex patterns (hereby referred to as complex MMN; cMMN) including descending-pitch tone pairs among standard ascending-pitch tone pairs (14–19), repeated tones among a pattern of alternating tones (20), rules that predict the duration or pitch of the next tone (21), missing tones in groups of identical tones (22), and extra tones that were identical to the other tones in a group (23), among other patterns. Rather than relying on an infrequent physical change among frequent identical sounds, cMMN depends on the abstraction of some sort of pattern rule. Complex auditory pattern tasks may elicit a later deviance-related negativity (i.e., cMMN) than the sMMN. Several studies have reported multiple MMNs, including the late cMMN, in response to a complex pattern deviant (24–28). We hypothesize that the later, presumably more computationally-demanding cMMN is impacted earlier in psychotic disease course.

Complex auditory pattern analysis and cMMN have been examined in long-term illness. Alain et al (20) found no significant difference in cMMN to a repeat deviant in long-term schizophrenia, although there was a trend-level group difference when the nose electrode was used as the reference. The cMMN to a missing tone on a gestalt grouping task was significantly reduced in long-term schizophrenia (29) and in early-course schizophrenia (30). The cMMN to infrequent extra tones in a gestalt grouping task is reduced in long-term schizophrenia (23). Descending pitch deviants among ascending pitch paired tones produced a smaller cMMN in those with long-term schizophrenia (31). The cMMN to tones that violate an ascending pitch pattern, and to tones that violate a musical scale pattern are reduced in schizophrenia (32). Thus, like sMMN, cMMN appears to be robustly reduced in long-term schizophrenia. However, cMMN tends to have a longer rather than later latency than sMMN, the similarities and differences in neural substrate between sMMN and cMMN are not known, and it has rarely been examined in close proximity to the emergence of psychosis (at first episode or earlier).

Having validated complex auditory pattern protocols as eliciting reduced cMMN in long-term illness, we have begun testing them in FESz individuals. Although descending pitch deviants among ascending pitch paired tones showed reduced cMMN in long-term schizophrenia, tone-pair cMMN was within normal limits in FESz (31). In the current study, we measured MMN to violations in an extra-tone gestalt grouping rule in SZ and in FESz, modified from a protocol validated to elicit reduced cMMN in Sz (23). Further, sMMN (pMMN and dMMN) were examined to compare effect sizes between various paradigms.

Although sMMN may not be highly sensitive to pathophysiology in early disease course, functional decline spans several domains (and predates the onset of overt psychosis). Keshavan and colleagues (personal communication, November 18, 2016)^1^ coined the acronym CLAASSIC for this progression, representing deficits first in Cognition and Learning, then problems in Affect and increased Anxiety, followed by Social deficits, and finally a progression from Subthreshold to Intermittent to Chronic psychotic symptoms. Similarly, Cornblatt et al. (33) coined the acronym CASIS for a progression of Cognitive deficits followed by Affective disturbance, Social dysfunction, increased Isolation, and finally increasing School and work impairments. Cornblatt et al (34) reported that social and role functioning were more impaired individuals later transitioning to psychosis compared to individuals that did not transition to psychosis. Thus, in addition to examination of cMMN amplitudes in FESz, we examined the degree to which cMMN reductions correlated with social deficits at first clinical contact.

## Methods

### Participants

Twenty-two FESz recruited from consecutive admissions to Western Psychiatric Institute and Clinic (WPIC) inpatient and outpatient services were compared with 22 healthy controls (HC). Fourteen FESz were diagnosed with schizophrenia (paranoid = 11, undifferentiated = 3), 2 with schizoaffective disorder (depressed subtype), 5 with psychotic disorder NOS, and 1 with schizophreniform disorder. FESz participated within two months of their first clinical contact for a first episode of psychosis, and had less than 2 months of lifetime antipsychotic medication exposure. Five FESz (22.7%) were unmedicated.

All subjects had normal hearing as assessed by audiometry, at least nine years of schooling, and an estimated IQ over 85. None of the participants had a history of concussion or head injury with sequelae, history of alcohol or drug addiction, detox in the last 5 years, or neurological comorbidity. Groups were matched for age, gender, premorbid estimates of intelligence based on the Wechsler Abbreviated Scale of Intelligence (WASI) IQ, and parental socioeconomic status. The 4-factor Hollingshead Scale was used to measure socioeconomic status (SES) in participants and in their parents. FESz evinced trend-level lower SES than HC, consistent with social and occupational impairment as a disease consequence (see **Table 1** for demographic measures). All participants provided informed consent, and were paid for participation. All procedures were approved by the University of Pittsburgh IRB.

**Table 1:** Demographic and clinical information.

	FESz	HC	p
**N (F)**	22 (7)	22 (9)	.53
**Age (sd)**	22.0 (4.8)	23.6 (7.8)	.42
**SES (sd)**	30.2 (13.1)	37.1 (13.0)	.09
**PSES (sd)**	43.8 (13.7)	49.5 (8.1)	.11
**PANSSP**	21.1 (4.8)		
**PANSSN**	17.6 (5.3)		
**PANSST**	77.7 (14.5)		
**SAPS**	1.7 (0.7)		
**SANS**	2.0 (0.6)		
**MEDS**	124.9 (165.7)		

SES, Socioeconomic status; PSES, Parental socioeconomic status; PANSSP, Positive and Negative Syndrome Scale positive factor score; PANSSN, PANSS negative factor score; PANSST, PANSS total score; SAPS, Scale for the Assessment of Positive Symptoms Averaged Global Scores; SANS, Scale for the Assessment of Negative Symptoms Averaged Global Scores; MEDS, Antipsychotic medication in chlorpromazine equivalents (oral dosages from Andreasen et al. (35), depot dosages from Gardner et al. (36).

#### Diagnostic Assessments

Diagnosis was based on the Structured Clinical Interview for DSM-IV (SCID-P). Symptoms were rated using the Positive and Negative Symptom Scale (PANSS), Scale for Assessment of Positive Symptoms (SAPS), and Scale for Assessment of Negative Symptoms (SANS). All tests were conducted by an expert diagnostician (see **Table 1** for clinical measures).

#### Neuropsychological Tests

All participants completed the MATRICS Cognitive Consensus Battery and the WASI. Social functioning was assessed with the Global Assessment Scale (GAS), Global Functioning: Social and Role scales (GF:S, GF:R), the brief UCSD Performance-based Skills Assessment (UPSA-B) and the Social Functioning Scales (SFS, see **Table 2** for neuropsychological test and social functioning scores).

**Table 2:** Neuropsychological and social functioning measures.

	FESz	HC	p
**WASI IQ**	108.1 (14.7)	106.3 (8.8)	.62
**MATRICS Speed**	45.5 (16.3)	50.6 (7.7)	.19
**MATRICS AttVig**	41.6 (12.0)	48.4 (6.3)	**.025**
**MATRICS WM**	44.9 (14.6)	45.5 (10.2)	.86
**MATRICS Verbal**	44.2 (12.2)	51.8 (10.2)	**.031**
**MATRICS Visual**	43.5 (12.4)	43.9 (7.5)	.88
**MATRICS Reason**	45.8 (11.8)	50.1 (6.8)	.15
**MATRICS SocCog**	43.1 (11.2)	54.1 (5.9)	**<.001**
**MATRICS Overall**	39.5 (14.5)	48.5 (7.1)	**.013**
**GAS**	35.6 (8.9)		
**UPSA-B Comm**	72.7 (13.2)		
**UPSA-B Finance**	82.6 (14.8)		
**UPSA-B Total**	77.7 (9.7)		
**GF : Role Current**	5.9 (2.2)	9.0 (0.2)	**<.001**
**GF : Role Highest**	7.7 (1.2)	9.0 (0.0)	**<.001**
**GF : Role Lowest**	5.7 (2.2)	9.0 (0.2)	**<.001**
**GF : Social Current**	5.4 (1.8)	9.0 (0.2)	**<.001**
**GF : Social Highest**	7.2 (1.2)	9.0 (0.2)	**<.001**
**GF : Social Lowest**	5.2 (1.7)	9.0 (0.1)	**<.001**
**SFS : Withdrawal**	100.6 (11.1)		
**SFS : Interpersonal**	121.77 (22.3)		
**SFS : Recreational**	110.7 (11.8)		
**SFS : Employment**	117.4 (5.4)		
**SFS : Independence-comp**	114.9 (10.1)		
**SFS : Independence-perf**	102.7 (12.0)		
**SFS : Pro-social**	109.7 (10.8)		

Values are mean (sd). Significant p values are **bolded**. WASI, Wechsler Abbreviated Scale of Intelligence; MATRICS, Measurement and Treatment Research to Improve Cognition in Schizophrenia Consensus Cognitive Battery; MATRICS Speed, Speed of processing t-score; MATRICS AttVig, Attention/vigilance t-score; MATRICS WM, Working memory t-score; MATRICS Verbal, Verbal memory and learning t-score; MATRICS Visual, Visual learning and memory t-score; MATRICS Reason, Reasoning and problem solving t-score; MATRICS SocCog, Social cognition t-score; MATRICS Overall, Overall t-score; GAS, Global Assessment Scale; UPSA-B, Brief UCSD Performance-based Skills Assessment; UPSA-B Comm, Communication subscore percentile; UPSA-B Finance, Finance subscore percentile; UPSA-B Total, Total score percentile; GF Role current/lowest/highest, Global Functioning : Role scale current (last month)/lowest past year/highest past year; GF : Social current/lowest/highest, Global Functioning Social scale current (last month)/lowest past year/highest past year; SFS : Withdrawal, Social Functioning Scale : Social engagament/withdrawal scaled score; SFS : Interpersonal, Social Functioning Scale : Interpersonal scaled score; SFS : Recreation, Social Functioning Scale : Recreation scaled score; SFS : Independence-comp, Social Functioning Scale : Independence-competence scaled score; SFS : Independence-perf, Social Functioning Scale : Independence-performance scaled score; SFS : Pro-social, Social Functioning Scale : Pro-social scaled score.

### Procedure

EEG was collected while subjects underwent two passive auditory tasks. Stimuli were generated with Tone Generator (NCH Software), and presented in Presentation (Neurobehavioral Systems, Inc.). Binaural auditory stimuli were presented at 80 dB using Etymotic 3A insert earphones, with loudness confirmed with a sound meter. Participants watched a silent nature video while tones were played over earphones. They were asked to concentrate on the movie and ignore the tones.

### Stimuli

#### Simple MMN

Stimuli comprised a standard tone (1 kHz, 50 msec duration, 5 msec rise/fall), a pitch deviant (1.2 kHz, 50 msec duration, 5 msec rise/fall), and a duration deviant (1 kHz, 100 msec duration, 5 msec rise/fall), presented with a stimulus onset asynchrony of 330 msec. A total of 1600 tones were presented, including 1,280 standards (80%), 160 pitch deviants (10%), and 160 duration deviants (10%).

#### Complex MMN

Temporal proximity was used to form discrete groups of tones, with a SOA within groups of 330 msec and an inter-trial interval of 750 msec. A total of 350 groups were presented. The standard group consisted of 3 identical tones (300 groups, 1 kHz, 50 msec duration, 5 msec rise/fall, identical to the standard tone in the simple MMN task). The deviant groups (50 groups, 14.3%) contained an additional fourth identical tone, and never immediately followed one another.

### EEG Recording

EEG was recorded from a custom 72 channel Active2 high impedance system (BioSemi), comprising 70 scalp sites including the mastoids, 1 nose reference electrode, and 1 electrode below the right eye. The EEG amplifier bandpass was DC to 104Hz (24 dB/octave rolloff) digitized at 512Hz, referenced to a common mode sense site (near PO1).

### MMN Analysis

Processing was done off-line with EEGLAB (37) and BrainVision Analyzer2 (Brain Products GMBH). First, using EEGLAB, EEG was filtered at 0.5 Hz to remove DC drifts and skin potentials. Data were visually examined and any channels with excessive noise were interpolated. AMICA was used to remove one vertical and one horizontal EOG component.

Following pre-processing in EEGLAB, event-related potentials were processed using BrainVision Analyzer2. Data were low-pass filtered at 20 Hz to remove muscle and other high frequency artifact, and rereferenced to averaged mastoids.

#### Simple MMN

Epochs of 350 msec, including a 50 msec prestimulus baseline, were extracted to deviant tones, and the standard tones preceding a deviant. Epochs were DC detrended between the first and last 50 msec, baseline corrected, and subsequently rejected if any site contained activity ±50 μV. Averages were constructed for the standard tones preceding a deviant, pitch deviants, and duration deviants. Pitch MMN (pMMN) was visualized by subtracting the standard average from the pitch deviant average. Duration MMN (dMMN) was visualized by subtracting the standard average from the duration deviant average. MMN was measured by detection of the peak MMN at FCz in the group grand averages, and voltage then averaged over a 25 msec window centered on that latency for each individual at 6 frontocentral sites (F1, Fz, F2, FC1, FCz, FC2) where cMMN was largest (pMMN: FESz 95–120 msec; HC(FE) 95–120 msec; Sz 95–120 msec; HC(Sz) 97–122 msec, dMMN: FESz 158–183 msec; HC(FE) 162–187 msec; Sz 168–193 msec; HC(Sz) 156–181 msec).

#### Complex MMN

Epochs of 750 msec were extracted, including a 100 msec prestimulus baseline. All epochs were DC detrended between the first and last 50 msec, baseline corrected, and subsequently rejected if any site contained activity ±50μV. Averages were constructed for the standard group last tones and the deviant group extra tones. cMMN was visualized by subtracting the standard group ending tone average from the deviant extra fourth tone average. MMN was measured by detection of the peak latency of the late cMMN at FCz in the group grand averages, and voltage then averaged over a 25 msec window centered on peak latency for each individual at the 6 frontocentral sites. Due to the apparent early and late MMNs, two time periods were analyzed; early cMMN (130–155 msec for all groups), and late cMMN (430–455 msec for all groups).

### Analyses

Group demographics and neuropsychological scores were compared using t-tests and chi-squared tests where appropriate. MMN analyses utilized repeated-measures ANOVA, with group (FESz, HC) as the between subjects factor, and electrode chain (F or FC) and site (left, central, or right) as within subjects factors. The Huynh-Feldt epsilon was used to correct for potential violations of sphericity of the site factor. Effect sizes were calculated as Cohen's d at FCz, the difference between the group means expressed in pooled standard deviations. Two-tailed Spearman's correlations were used to examine relationships between MMN at FCz and demographic, clinical, and neuropsychological items. Values are reported as Mean ± SD. Significance was attained at p < .05.

## Results

### Simple MMN

**Figure 1:**
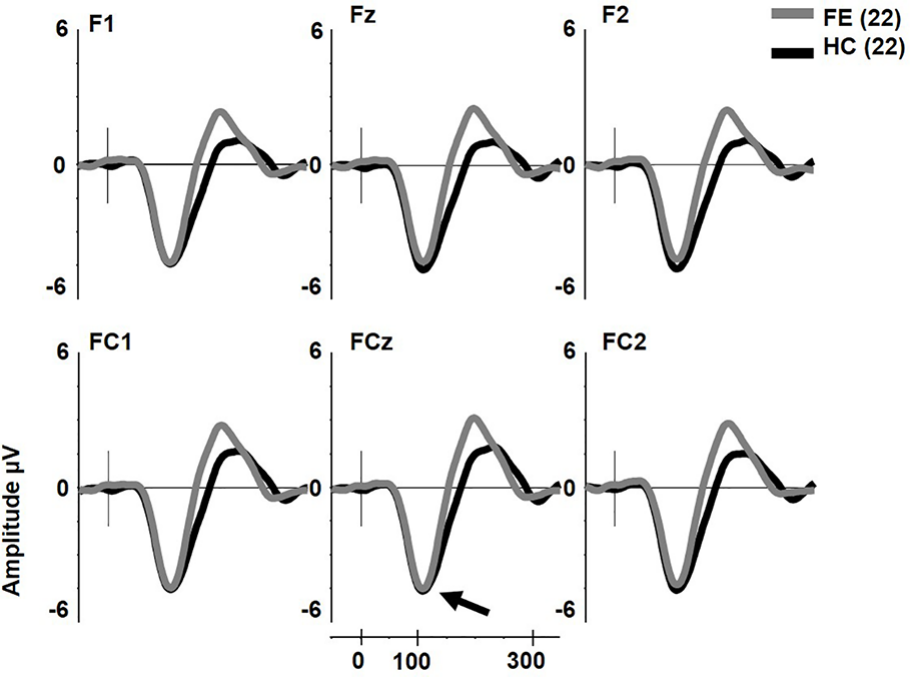
Pitch deviant mismatch negativity (MMN) in first psychotic episode in the schizophrenia-spectrum (FESz) and healthy control (HC) (arrow). Note that FESz are well within the normal range in pMMN at the group level.

pMMN was not reduced in FESz (F1,42 = 0.2, p = .657, **Figure 1**). No other main effects or any interactions were significant. The effect size d for pMMN at FCz was 0.08 SD (See **Table 3** for individual site voltages).

**Table 3:** Simple mismatch negativity (sMMN) and complex MMN (cMMN) values.

	F1	Fz	F2	FC1	FCz	FC2
**pMMN**						
**FESz**	-4.2 (1.7)	-4.1 (1.7)	-4.0 (1.7)	-4.2 (1.7)	-4.2 (1.7)	-4.1 (1.7)
**HC**	-4.3 (1.6)	-4.5 (1.6)	-4.5 (1.7)	-4.3 (1.8)	-4.4 (1.9)	-4.4 (2.0)
**dMMN**						
**FESz**	-3.2 (1.8)	-3.3 (1.8)	-3.4 (1.8)	-3.3 (1.9)	-3.5 (1.9)	-3.5 (1.8)
**HC**	-3.2 (2.4)	-3.4 (2.2)	-3.4 (2.3)	-3.4 (2.5)	-3.5 (2.7)	-3.5 (2.6)
**cMMN**						
**FESz**	-0.1 (1.6)	-0.1 (1.5)	-0.2 (1.4)	0.0 (1.5)	0.0 (1.8)	-0.2 (1.4)
**HC**	-1.1 (1.6)	-1.1 (1.6)	-1.3 (1.6)	-1.2 (1.5)	-1.4 (1.7)	-1.3 (1.6)

Values are mean (SD). Values are in μV.

**Figure 2:**
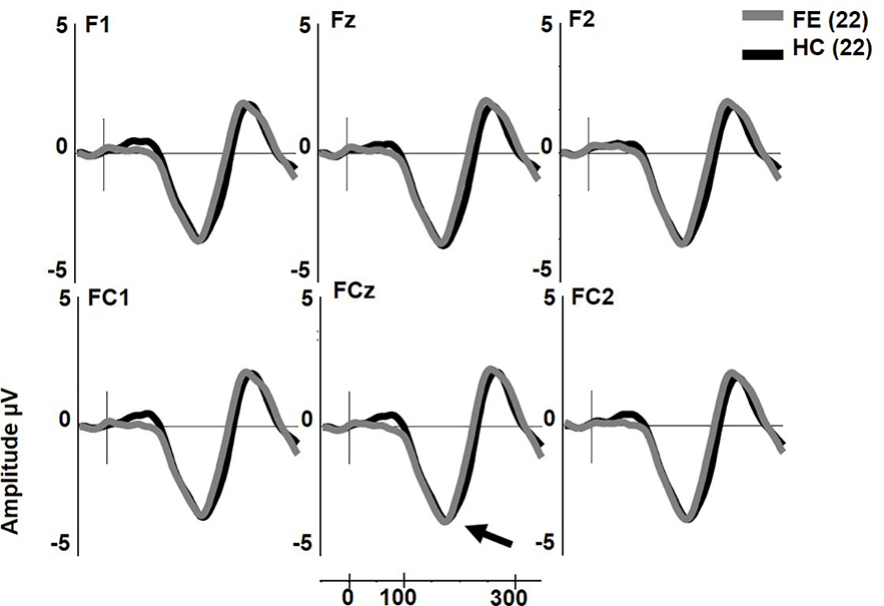
Duration deviant mismatch negativity (MMN) in first psychotic episode in the schizophrenia-spectrum (FESz) and healthy control (HC) (arrow). Note that FESz are well within the normal range in pMMN at the group level.

dMMN was not reduced in FESz (F1,42 = 0.001, p = .976, **Figure 2**). dMMN was marginally larger along the FC chain than the F chain (F1,42 = 3.62, p = .064) and this distribution did not differ between groups. dMMN was larger at center and right sites than the left (F2,84 = 8.93, p = .001, ϵ = 0.80) and this distribution did not differ between groups. No remaining interactions were significant. The effect size d for dMMN at FCz was -0.02 SD, indicating FESz were larger than HC, albeit non-meaningfully (See **Table 3** for individual site voltages).

### Extra-Tone Complex MMNs

**Figure 3:**
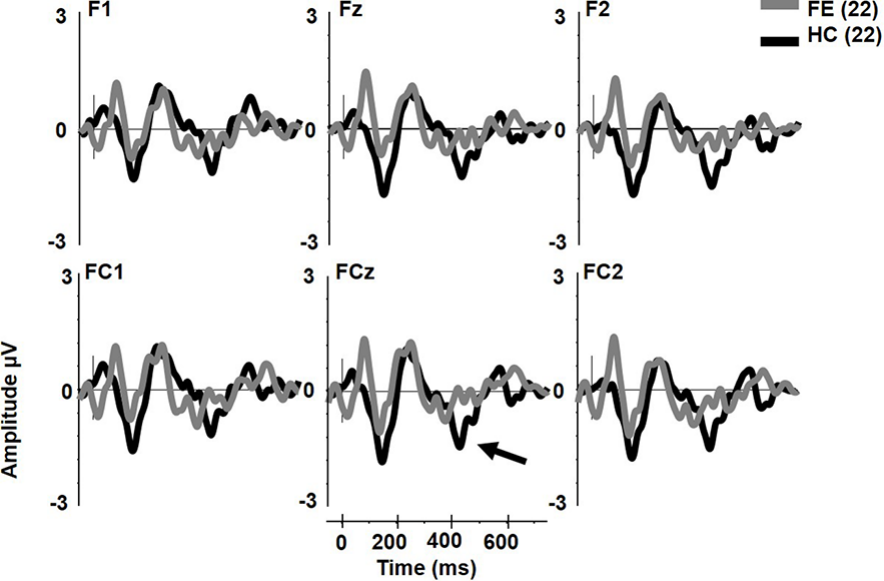
Extra tone gestalt grouping rule complex mismatch negativity (MMN) in first psychotic episode in the schizophrenia-spectrum (FESz) and healthy control (HC). Note the lack of late cMMN in FESz (arrow).

The early cMMN was not different between groups (p > .2) and will not be discussed further. Unlike pMMN and dMMN, which were not reduced, late cMMN (henceforth referred to as cMMN) was significantly impaired in FESz (FESz: -0.1 μV, HCFE: -1.2 μV. F1,42 = 6.2, p = .016. **Figure 3**. See **Table 3** for site voltages). The Cohen's d for group differences at FCz was 0.82 SD. No other main effects or interactions were significant.

### Neuropsychological and Clinical Correlations

**Figure 4:**
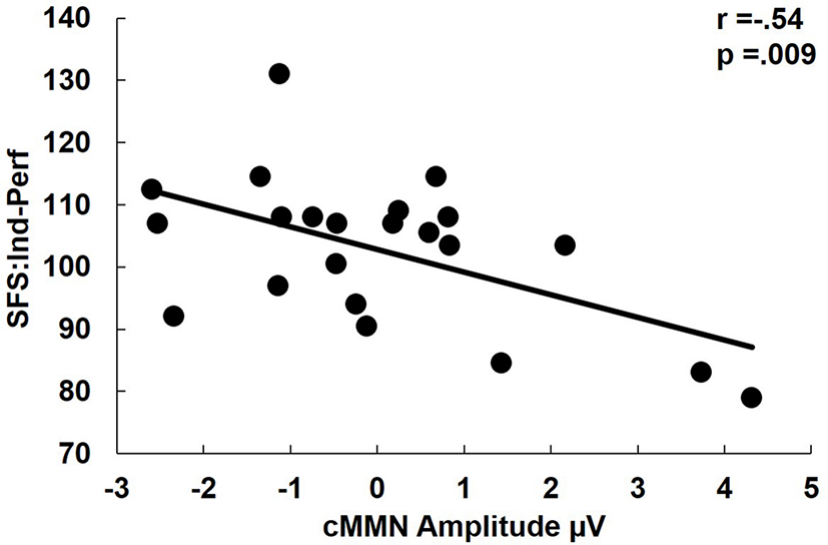
Correlation of complex mismatch negativity (cMMN) with Social Functioning Scales Independence-performance scaled scores. Independence-performance measures the use an attained skill for successful social functioning. Hence, lower scores indicate the failure to use an available skill, rather than a lack of social skills per se. Note that among first psychotic episode in the schizophrenia-spectrum (FESz) smaller cMMN is a reliable index of a failure to successfully utilize one's full set of social skills. Note: cMMN measured from FCz.

There were no significant correlations in FESz between cMMN amplitudes and WASI or MATRICS composite scores. Likewise, there were no significant correlations with PANSS total scores or positive, negative, or thought disorder factor scores. Among social functioning measures, larger cMMN was associated with higher SFS independence-performance scaled scores (r = -.54, p = .009, **Figure 4**). cMMN was uncorrelated with duration of untreated psychosis (DUP).

## Discussion

In this sample of individuals at first contact with psychiatric services for psychosis, with only a short course of antipsychotic medication, sMMN was not reduced at the group level, with a small effect size for both pMMN (d =0.08) and dMMN (d =-0.02). By contrast, the later cMMN to a pattern deviant, in this case an extra 4^th^ tone among groups of 3 tones, revealed a significant and large deficit (d =0.82). Thus, the results are consistent with the hypothesis that the increased computational demand of pattern processing as reflected in cMMN by contrast with the relatively easily discriminable physical parameter changes eliciting sMMN is more sensitive to the earlier and more subtle pathophysiology during the early course of the progressive pathology in schizophrenia. Although the exact neural correlates of the cMMN are unknown, it is assumed the same circuits process novelty for sMMN and cMMN within the prediction error scheme (38), and likely rely on the same NMDAr mediated circuit plasticity (39). This may be a promising framework for development of a biological circuit-based approach to new pharmacologic interventions, but the present data do not allow extrapolation to the circuit or synaptic level. Future source-resolved human studies and invasive animal work may shed light on the regional and circuit similarities and differences in the cortical substrate of cMMN and sMMN.

cMMN was not significantly different from zero in FESz. Hence, it is not entirely clear if correlations are valid, in the sense that the cMMN may only reflect neurophysiological noise. However, FESz did show a range of cMMN amplitudes (**Figure 4**), and those with larger cMMN tended to have better utilization of social skill sets. While cMMN has not been correlated with social functioning in FESz previously, we have shown that cMMN correlated with emotional withdrawal, poor attention, and lack of spontaneity and flow of conversation in long term schizophrenia (23). The strong association between reduced cMMN and impaired utilization of available social skills is consistent with the original reports of reduced social functioning being associated with dMMN in long term schizophrenia, e.g., (40). Still, given the relatively restricted range of cMMN this finding would need to be replicated and investigated further for meaningful interpretation.

The fact that cMMN was not significantly different from zero in FESz argues strongly for its utility as a biomarker of disease presence. This is even more compelling in the context of an essentially normal pMMN and dMMN in the same FESz subjects that showed a markedly impaired cMMN. Thus, it appears that pattern-deviance tasks that necessitate a higher level of processing may be more sensitive to subtle auditory cortex pathology than relatively simple changes in a single acoustic feature. One might speculate that the degree of pathology needed to impair detection of pure tone differences is rather substantial. cMMN appears to be sensitive to more modest pathology. Future studies should compare first episode schizophrenia-spectrum to affective-spectrum individuals to determine the specificity of the cMMN deficit on this task.

Although CHR individuals were not tested, we suggest a measure with a large deficit in FESz may be useful for investigation of CHR individuals. While all CHR individuals may show general auditory pathology, it is possible that the cMMN deficits emerge only in those that are in the true prodrome to schizophrenia. While no single test is likely to be diagnostic, the extra tone cMMN appears to be an important tool that when used in conjunction with other biological, neuropsychological, and clinical measures can provide an objective indication of risk for transition to psychosis. It is important to note that most “risk calculators” for transition to psychosis and studies of factors predisposing to eventual conversion are based on clinical and psychological rather than biological aspects of presentation. For example, Cannon et al. (41) found 5 factors contributed to risk for transition to psychosis: genetic risk with deterioration in functioning, greater paranoia and unusual thought content, worse social functioning, and a history of substance abuse. Cannon et al. (42) reported that greater paranoia and unusual thought content, greater decline in social functioning, poor verbal learning and memory, slow speed of processing, and younger age at clinical contact contributed to risk for transition to psychosis. Ruhrmann et al. (43) developed a prediction model based on schizotypal disorder, positive symptoms, bizarre thinking, sleep disturbances, decline in social functioning in the last year, and years of education. Fusar-Poli et al. (44) have developed an online calculator for the transition to psychosis among individuals accessing secondary mental health care (not the general population), based on presumptive diagnosis, age, sex, sex by age, and ethnicity. None of these calculators include a biological measure.

By contrast with the clinically-based risk calculators, a growing number of studies explored baseline and longitudinal measures useful in predicting group membership once transition to psychosis occurred (or more correctly over a followup period such as 2, 3, or 5 years). In their recent meta-analysis, Studerus, Ramyead, and Riecher-Rössler (45) indicated 91 studies that developed or validated a prediction model for the transition to psychosis. Among those studies, only 10 used EEG measures, 7 used MRI measures, and 5 used blood-based biomarkers. Thus, objective biological tests that may indicate the transition to psychosis are a critical unmet need for early intervention. We do not know if cMMN will be a useful biomarker before the onset of psychosis, but its reduction at first psychosis with a large effect size is encouraging. Future work needs to examine the possibility in CHR individuals that those in a true psychosis prodrome show selective cMMN reduction. This presupposes that the same auditory pathophysiology is common across various etiologies to psychosis. As described earlier, the fact that most individuals in the early and long-term phases of schizophrenia show MMN reduction suggests it is a systems-level pathology that cuts across specific etiologies for schizophrenia.

Several issues remain unanswered. Although the effect size for cMMN reduction was large (d = 0.82), the samples were relatively modest, and the results must be replicated. Because this is the first application of the extra tone cMMN task at first episode, and there are relatively few cMMN studies in psychosis, the reliability of the measure is unknown. The cMMN was small in HC, which may limit its utility. Currently, we are developing new pattern MMN paradigms to elicit larger cMMN in HC, which presumably will increase the group separation. We do not know whether the cMMN and sMMN share the same biological substrate, particularly given the longer latency of cMMN. Neither do we know what the distributed neural system underlying cMMN is. Relatedly, we do not know whether cMMN is dependent on NMDAr function. Future work must resolve these molecular issues. It is not known what cMMN looks like in CHR. A robust decrement in cMMN at first episode in the lack of sMMN reduction indicates that the probability of cMMN providing useful information about subtle underlying neural pathophysiology in true prodromal cases among CHR individuals is greater than sMMN. Still, it remains unknown. Currently, we are testing this paradigm in such individuals.

In summary, we showed that cMMN to an extra tone among frequent groups of standard size was reduced in FESz, while pMMN and dMMN were not. Further the cMMN correlated with social functioning, with worse functioning associated with smaller cMMN. These novel data provide the first step in development of a MMN-based task that is more sensitive to the subtle pathology early in the course of schizophrenia. We speculate that cMMN may be a useful probe of pathophysiology in the true psychosis prodrome.

## Data Availability Statement

The raw data supporting the conclusions of this article will be made available by the authors, without undue reservation, to any qualified researcher.

## Ethics Statement

The studies involving human participants were reviewed and approved by University of Pittsburgh Institutional Review Board. The patients/participants provided their written informed consent to participate in this study.

## Author Contributions

DS provided substantial contributions to the conception and design of the work; the acquisition, analysis, and interpretation of data for the work; and drafting the work and revising it critically for important intellectual content. BC provided substantial contributions to the acquisition, analysis, and interpretation of data for the work, and revising the report critically for important intellectual content. SH provided substantial contributions to the acquisition, analysis, and interpretation of data for the work, and revising the report critically for important intellectual content. All authors provided approval for publication of the content, and agree to be accountable for all aspects of the work in ensuring that questions related to the accuracy or integrity of any part of the work are appropriately investigated and resolved.

## Funding

This work was supported by NIH R01 MH094328 to DS.

## Conflict of Interest

The authors declare that the research was conducted in the absence of any commercial or financial relationships that could be construed as a potential conflict of interest.

## Footnotes

1 At the Annual Pittsburgh Schizophrenia Conference.
